# Supplementary material for: Learning curve and surgical outcome of robotic assisted colorectal surgery with ERAS program
Source: Sci Rep. 2022 Nov 29;12:20566. doi: 10.1038/s41598-022-24665-w (PMC9709162; doi:10.1038/s41598-022-24665-w)
Supplement: Supplementary file 2 — Supplementary Information 2. [file 41598_2022_24665_MOESM2_ESM.zip › RAL Raw data-Ñ[▒K/126/Patho_ñw╝╨░O▒Kñσ.pdf]

臺中榮民總醫院 臺中市臺灣大道四段1650號 病理部活體組織切片 臨床醫學實驗及病理診斷中心 檢驗(查)報告

[redacted]

Pathologic diagnosis:
1. Rectum, robotic low anterior resection --- Status post neoadjuvant therapy with chronic inflammation and fibrosis. No residual tumor seen. The bilateral cut ends and perirectal lymph nodes (0/12) are unremarkable.
2. IMA root, dissection --- Unremarkable (0/2).

Prognostic and predictive factor:
1. Tumor size: No residual tumor.
2. Depth of invasion: No evidence of primary tumor.
3. Histologic grade: Not applicable.
4. Angiolymphatic invasion: Not identified.
5. Perineural invasion: Not identified.
6. Tumor deposits (discontinuous extramural extension): Not identified.
7. Macroscopic tumor perforation: Not identified.
8. Surgical Margins: a) Proximal Margin: Uninvolved. b) Distal Margin: Uninvolved. c) Radial or Mesenteric Margin: Uninvolved.
9. Lymph node status: Uninvolved (0/14).
10. Extranodal involvement: Not applicable.
11. Tumor regression grading S/P CCRT: Present, no viable cancer cells (complete response, score 0).
12. Type of polyp in which invasive carcinoma arose: Not applicable.
13. Additional pathologic findings: Not identified.
14. Pathological TNM Stage: ypT0N0(According to the eighth edition, American Joint Committee on Cancer Staging Guidelines for Tumors).
15. TNM descriptors: y (Post-treatment).

Gross description:
The specimen consists of 1) a segment of colon, 10.5 cm in length and 4.5 cm in proximal cut end circumference, 5.5 cm in distal circumference, and perirectal soft tissue. An ulcerative lesion, 3.5x2.5 cm, is noted, very close to the distal cut end. On cross section, the lesion is fibrotic, involving whole layer of rectum, very close to the radial margin. The proximal cut end is free of tumor grossly. 2) IMA root, a piece of tan yellow soft tissue, 3x2x0.7 cm. Representative part for section: A) proximal cut end B-M) tumor with distal cut end (yellow ink) and radial cut end (blue ink) P) specimen 2 Q-S) perirectal lymph nodes.
#T-59600_2 #M-09413_2 0 1105 00SP00
#T-C4560_2 #M-09183_2 0 1105 000000

已電子簽章

敬告：本單僅供醫師參考使用，不具任何申請效力！！
請病患妥為保管，若不慎遺失需自負個人資料外洩責任！
